# Metacontrast masking of symmetric stimuli

**DOI:** 10.1371/journal.pone.0330019

**Published:** 2025-08-07

**Authors:** Giulio Contemori, Marianna Musa, Carolina Maria Oletto, Stefano Vicentin, Luca Battaglini, Giorgia Cona, Marco Bertamini

**Affiliations:** 1 Department of General Psychology, University of Padova, Padova, Italy; 2 Padua Neuroscience Center, University of Padova, Padova, Italy; Education University of Hong Kong, HONG KONG

## Abstract

This study investigated whether symmetry perception is vulnerable to metacontrast masking and whether such masking selectively disrupts feedback-dependent visual processes. Across four experiments, we employed a metacontrast paradigm with briefly presented targets (20 ms) followed by masks at varying stimulus onset asynchronies (SOAs), manipulating both target–mask configuration and task demands. All experiments produced the classic U-shaped accuracy-by-SOA curve associated with Type B masking, where performance is lowest at intermediate SOAs. Critically, performance at 0 ms SOA varied depending on the perceptual compatibility of the stimuli. In Experiments 1 and 2, the target and mask were spatially complementary and could be perceptually grouped into a unified figure. Under these conditions, performance at 0 ms SOA exceeded the no-mask baseline, reflecting facilitation due to perceptual integration. In contrast, in Experiments 3 and 4—where the stimuli and mask had no complementary shape and could not be integrated into a coherent object—performance at 0 ms SOA was slightly suppressed, indicating that integration failed to occur. These findings suggest that facilitation at short SOAs depends on the rapid formation of a coherent perceptual object, whereas symmetry detection—requiring temporally extended, feedback-supported integration—is more susceptible to early interruption by masking. Together, these results support both dual-channel and recurrent models of visual masking. Type B suppression reflects interactions between fast feedforward and slower feedback signals, while the presence or absence of early facilitation serves as an index of perceptual organization. These findings underscore how stimulus structure and task context affect the temporal dynamics of shape perception.

## Introduction

Metacontrast masking is a form of backward visual masking in which the visibility of a briefly presented target is diminished or abolished by a subsequently presented mask that does not spatially overlap with the target. One of the defining features of this phenomenon is the characteristic inverted U-shaped function relating masking strength to stimulus onset asynchrony (SOA), commonly referred to as Type B masking. Suppression is typically maximal at intermediate SOAs (approximately 20–100 ms), with visibility improving at both shorter and longer delays [1]. In some cases, particularly when the target and mask are spatially or perceptually complementary—such that they form a coherent object when presented simultaneously—target visibility has been observed to improve rather than decline at short SOAs (0–20 ms) [2–4].

The non-monotonic relationship between SOA and perceptual suppression was first documented in seminal behavioural work by Werner (1935) [5] and Burchard & Lawson (1973) [6], who proposed that cortical inhibition plays a central role in masking dynamics. Subsequent neurophysiological studies provided converging evidence. For example, Kondo & Komatsu (2000) [7] demonstrated that the introduction of a backward mask significantly reduced neuronal responses to a target stimulus in macaque area V4—a mid-level visual region associated with form processing. Notably, this suppression occurred even when the mask and target were spatially non-overlapping, suggesting temporally constrained interactions beyond early retinotopic input channels. These findings underscore the involvement of higher-order visual areas in metacontrast masking, consistent with more recent evidence linking V4 and other extrastriate regions (e.g., LOC) to global form perception and symmetry processing [8]. Despite decades of research across behavioural, physiological, and computational domains, the precise mechanisms underlying metacontrast masking remain actively debated [9–12].

Three principal theoretical frameworks have been proposed to account for the phenomenon. The first, grounded in feedforward models of visual processing, attributes masking to lateral inhibition within early cortical areas. In this framework, the presentation of a mask activates inhibitory interneurons that suppress neural responses associated with the target, particularly at the spatio-temporal boundaries of its representation in primary visual cortex [13,14]. The model is particularly effective in explaining Type A masking functions, characterized by strong suppression at short SOAs that diminishes monotonically as temporal separation increases.

A second, increasingly influential model shifts the locus of suppression from feedforward circuits to cortico-cortical feedback loops. According to this feedback-based account, the initial activation of the visual system by the target proceeds without substantial interference from the mask; however, the mask interrupts the reentrant signalling necessary to stabilize and integrate the target’s representation into a coherent percept [15–18]. In this view, feedback from higher-tier visual areas such as the lateral occipital complex (LOC) or parietal cortex to early visual areas like V1 and V2 is essential for conscious perception. If this recurrent signalling is disrupted—particularly during the critical SOA window associated with Type B masking—the integration of visual information is impaired, resulting in perceptual suppression [19,20].

Neurostimulation and electrophysiological studies have provided compelling support for this feedback account. Ro et al., (2003) [21] demonstrated that transcranial magnetic stimulation (TMS) applied to the right lateral occipital cortex (approximately corresponding to area LOC) during the masking interval can restore visibility to otherwise suppressed targets. This finding suggests that the perceptual deficit in masking arises from disrupted reentrant processing in extrastriate areas, rather than from degraded early feedforward input. Notably, LOC has been strongly implicated in symmetry perception across fMRI, ERP, and TMS studies [22,23], indicating a potential convergence between the neural mechanisms of masking and those required for global form perception. Fahrenfort et al. (2007) [24] further showed that early ERP components (e.g., P1), typically associated with initial feedforward responses, remain intact under masking, whereas later components (e.g., P3), associated with reentrant processing and visual awareness, are selectively eliminated. Aydin et al., (2021) [3] provided additional support by demonstrating that masking effects depend on the polarity relationship between target and mask: when polarity is matched, masking follows a Type B pattern and coincides with the suppression of late (>250 ms) EEG components, again implicating feedback-dependent processes. Similarly, Railo & Koivisto, (2009) [25] observed that backward masking disrupts late-stage ERP components such as the Visual Awareness Negativity (VAN) and P3b, both of which are closely linked to conscious visual experience and are thought to depend on feedback-driven integration.

A third framework—the dual-channel model—offers a hybrid explanation that reconciles aspects of both feedforward and feedback-based accounts. Originally formulated by Breitmeyer & Ganz, (1976) [18] and elaborated by Breitmeyer & Öğmen (2006) [26], this model posits the existence of two parallel visual processing streams: a fast, transient channel associated with the magnocellular pathway, and a slower, sustained channel associated with the parvocellular pathway. The mask, processed more rapidly through the transient channel, can suppress or override the slower-developing representation of the target processed through the sustained channel—a mechanism known as interchannel inhibition. This interaction is particularly salient when the target and mask share the same contrast polarity and engage distinct processing streams, yielding the classic inverted U-shaped Type B function. In contrast, when both stimuli are processed within the same stream—such as when their contrast polarity differs—intrachannel inhibition dominates, resulting in a monotonic Type A suppression profile [3].

Crucially, the dual-channel model offers a flexible framework in which masking profiles emerge from the interplay of timing, contrast polarity, and stream-specific processing. It explains both early feedforward inhibition and later feedback suppression, particularly in tasks involving global integration. Yet, despite its breadth, empirical consensus remains elusive, and accumulating evidence increasingly supports feedback-based accounts.

Notably, the majority of prior investigations into metacontrast masking have employed highly simplified stimuli—such as disks, annuli, or bars [27–29]—designed to isolate low-level inhibitory processes. While such paradigms offer experimental control, they may not generalize to perceptual tasks that demand higher-order integration. A particularly important methodological feature of these studies is the frequent use of target–mask pairs that are spatially or perceptually complementary. For instance, a ring-shaped mask often surrounds a disk target in a manner that invites perceptual grouping. Such complementarity has been shown to facilitate integration of the two elements into a unified percept, especially when presented simultaneously (SOA = 0 ms). This integration can yield a paradoxical improvement in visibility—commonly referred to as facilitation—rather than suppression, an effect attributed not to the absence of masking but to the perceptual “pop-out” of a coherent shape [30–32]. Consequently, facilitation at short SOAs may obscure the suppressive dynamics that backward masking is intended to reveal, complicating efforts to disentangle feedforward and feedback contributions.

To overcome this confound, the present study incorporated both complementary (integrable) and non-complementary (non-integrable) target–mask configurations across experiments, allowing for a more principled examination of masking dynamics. Importantly, the study leverages symmetry perception as a testbed for probing feedback-dependent visual processing. Symmetry perception requires the integration of globally distributed features into a coherent perceptual whole [33,34], making it uniquely well-suited to reveal the role of reentrant mechanisms in masking paradigms.

Unlike simple shape detection—which can often be accomplished via localized, feedforward mechanisms—detecting bilateral symmetry involves mid- and high-level visual areas such as V3, V4, and the lateral occipital complex (LOC), as consistently demonstrated by both fMRI and ERP studies [22,35,36]. Although feedforward processing may suffice under ideal conditions—such as high-contrast, centrally presented stimuli of sufficient spatial extent [37,38]—a substantial body of research indicates that symmetry perception is highly context-sensitive and often relies on feedback mechanisms. Under degraded conditions, elevated task demands, or perceptual ambiguity, feedback becomes essential for resolving global structure [39, 40].

Psychophysical studies support this view. When symmetry stimuli are embedded in complex or low-salience arrays, they typically fail to elicit automatic pop-out effects and instead require serial or attentionally mediated search [41,42]. This is corroborated by Moreau et al. (2025) [43], who found no evidence of parallel processing even in optimally regular, high-symmetry textures. Instead, search efficiency was modulated by both symmetry type and texture regularity, with reflection and regularity each independently enhancing performance. Crucially, the absence of true pop-out even under favourable conditions suggests that symmetry detection depends on integrative processes more consistent with feedback than with feedforward feature detectors.

Convergent evidence comes from computational modelling. Sundaram et al. (2022) [44] showed that deep convolutional neural networks (DCNNs) lacking recurrent connections fail to generalize symmetry detection to novel exemplars, even when enhanced architectures are employed. By contrast, networks incorporating recurrence—functionally analogous to cortical feedback—successfully learned and generalized symmetry relations across diverse inputs. These findings highlight the computational necessity of recurrence for solving the spatial integration challenges inherent in symmetry perception.

Neurophysiological data further substantiate this framework. The Sustained Posterior Negativity (SPN), an ERP component that typically emerges 250–350 ms post-stimulus, is robustly associated with symmetry perception and is thought to reflect post-perceptual integration in extrastriate cortex [45]. Dering et al. (2024) [46] found that the SPN is significantly attenuated when symmetry is occluded, degraded, or task-irrelevant—conditions known to interfere with feedback-dependent processing. [47] similarly reported that SPN lateralization, a marker of hemispheric specialization for symmetry, arises only when participants are explicitly instructed to assess symmetry, indicating that top-down attentional control modulates reentrant integration. Additional support comes from [48], who employed frequency-tagged EEG to demonstrate that symmetry-specific neural responses are amplified when stimuli are task-relevant and attended. These findings suggest that attention enhances symmetry-related processing through top-down modulation, consistent with the engagement of feedback mechanisms.

This interpretation is further corroborated by neurostimulation studies. Cattaneo et al. (2011) [23] and Bona et al. (2014) [49] demonstrated that TMS applied to LOC disrupts symmetry discrimination, even when early sensory input remains unaffected. These results provide causal evidence for the involvement of higher-order visual areas and further implicate feedback as a necessary mechanism in symmetry perception.

The present study was designed to promote the recruitment of feedback mechanisms by imposing stringent temporal and perceptual constraints. Using a classical metacontrast masking paradigm with brief (20 ms) target exposures and variable stimulus onset asynchronies (SOAs)—in line with a recent protocol [3]—the task was optimized to limit the efficacy of purely feedforward processing and necessitate reentrant integration. The 20 ms exposure duration is particularly taxing for symmetry tasks, as most SPN paradigms employ much longer durations [45]. Extensive pilot testing was used to calibrate task difficulty, maintaining accuracy near 80% to ensure that both early sensory encoding and late-stage integration processes were engaged.

Finally, the neural architecture supporting symmetry perception is closely tied to the ventral visual stream. Global form analysis, spatial integration, and perceptual grouping—core functions of symmetry processing—are consistently localized to extrastriate regions such as V3, V4, and LOC, with minimal involvement from V1 [37,50]. This hierarchical organization reinforces the view that symmetry is not a low-level feature but rather an emergent property arising from recurrent interactions across higher-order visual areas.

Critically, these same ventral structures are also implicated in metacontrast masking. Functional imaging studies have shown that backward masking selectively disrupts activation in LOC and V4 [51,52]. Complementing these findings, laminar electrophysiological recordings in macaques have demonstrated that backward masking abolishes late activity in the supragranular and infragranular layers of V1—layers associated with cortico-cortical feedback—while sparing the early feedforward response in the granular layer 4 [15,16,53]. Van Kerkoerle et al. (2014) [54] further showed that alpha-band oscillations, typically associated with top-down feedback, are diminished in superficial layers during masking, while gamma-band activity in granular layers—reflecting feedforward input—remains largely intact. These temporal and laminar dissociations suggest that masking disrupts delayed, cortico-cortical feedback necessary for perceptual integration, while leaving initial feedforward processing unaffected. Taken together, these findings support the hypothesis that recurrent interactions between V1 and higher-order visual areas are essential for the construction of complex perceptual representations such as symmetry.

This neural profile offers a theoretically instructive test case for the dual-channel model of masking [18,26], symmetry detection—given its reliance on sustained, ventral-stream processing—should be especially susceptible to interchannel masking when preceded or followed by a transient, magnocellular mask. Accordingly, our paradigm offers a critical test of whether Type B masking can emerge from such cross-stream interactions during symmetry tasks. This prediction contrasts with claims made by Van Der Vloed et al. (2007) [55], who argued that symmetry perception is relatively immune to masking. However, their experimental paradigm diverged meaningfully from classical metacontrast designs. Specifically, their stimuli consisted of Gaussian-blob textures presented asynchronously in central and peripheral regions, aiming to test temporal segmentation rather than visual masking per se. While their results suggested that symmetry processing is temporally robust, their design lacked the spatial adjacency and transient onset features that define metacontrast masking. As such, their findings cannot be straightforwardly interpreted as evidence against masking effects on symmetry.

The present study addresses this gap by directly assessing whether symmetry perception—particularly when reliant on feedback-dependent integration—is susceptible to classical metacontrast masking. We conducted four experiments designed to systematically manipulate two critical dimensions: (1) the perceptual relevance of symmetry to the task, and (2) the spatial compatibility between target and mask. This approach enabled us to evaluate whether the resulting masking profiles followed Type A, Type B, or exhibited minimal suppression under varying demands for global integration.

Experiment 1 replicated the protocol of Aydin et al. (2021) [3], employing simple disk targets and annular masks to establish a baseline masking function in a low-level shape discrimination task. Experiment 2 introduced implicit symmetry via targets composed of disks with one or two missing segments; although symmetry was not explicitly task-relevant, prior research indicates that such stimuli can engage automatic symmetry processing mechanisms [56,57]. In Experiment 3, symmetry became an explicit task demand, as participants discriminated between symmetric and asymmetric octagons. Finally, Experiment 4 maximized the need for global integration by using abstract, irregular figures in which symmetry constituted the only diagnostic cue—thus minimizing the contribution of local features and foregrounding the role of reentrant visual processing.

## Methods

The metacontrast paradigm used was based on Aydin et al. (2021) [3], with two modifications: participants had no response time limit, both target and mask had the same polarity and were black against a mid-grey background. Each trial began with a red fixation cross (0.6° visual angle, 0.2° central dot) presented for 1000 ms. A target appeared for 20 ms, followed by the annular mask. We used nine levels of SOA between target and mask: 0, 10, 20, 40, 60, 80, 120, 160, and 200 ms. A no-mask condition was also present. The target was positioned 3° above the fixation cross, and the mask surrounded the target without spatial overlap. At 0 ms SOA, the target and mask were presented simultaneously for 20 ms. At 10 ms SOA, the target appeared alone for 10 ms, followed by 10 ms of overlap with the mask, which then lasted for another 10 ms. Participants responded after mask offset, using arrow keys.

Stimuli were generated using PsychoPy3 [58] and presented on an LCD monitor (1920x1080 pixels, 100 Hz). Participants were seated 57 cm from the screen in a quiet, dimly lit room, with head stabilization. Eye movements were monitored using a Gazepoint GP3 eye tracker, and stimuli were only presented when fixation was within ±1° of the screen centre.

Before the main experiment, participants completed 20 practice trials identical to the experimental trials, with feedback. In the main experiment, participants completed 200 randomized trials (20 per SOA condition), without feedback.

Experiment 1: Single-Segment Disk (Shape discrimination). The target stimulus was a white disk with a diameter of 2° visual angle. A 40° segment (an area between a chord and the corresponding arc) was removed from either the left or right side. The straight boundary of the removed segment (the chord) had a length of approximately 0.684° of visual angle with a sagittal height of 0.15°. The mask was a white ring with a minimal spatial separation of approximately 0.05° from the edge of the disk. Participants were asked to indicate whether the missing segment was on the left or right side of the disk.

Experiment 2: Double-Segment Disk (Implicit symmetry discrimination). The target was a disk, with two possible configurations: symmetric, with two 40° segments missing on opposite sides, or asymmetric, with only one segment missing. The task was to determine whether the target was missing one segment (asymmetric) or two segments (symmetric). The mask was the same as in Experiment 1.

Experiment 3: Irregular Polygons (Symmetry discrimination). The target was an irregular octagon generated by displacing the vertices of a regular octagon. Each octagon had eight vertices spaced at equal 45° intervals, and the displacement of each vertex varied between 0.1° and 0.3°, creating asymmetric shapes. In the symmetric condition, one half of the shape was mirrored along a vertical axis. Due to the octagonal structure, symmetric shapes contained more than one axis of symmetry. The average target size was 1.5° visual angle, and the mask surrounded the polygon with a separation of ~0.5° from its outermost points. Participants judged whether the polygon was symmetric or asymmetric.

**Fig 1:**
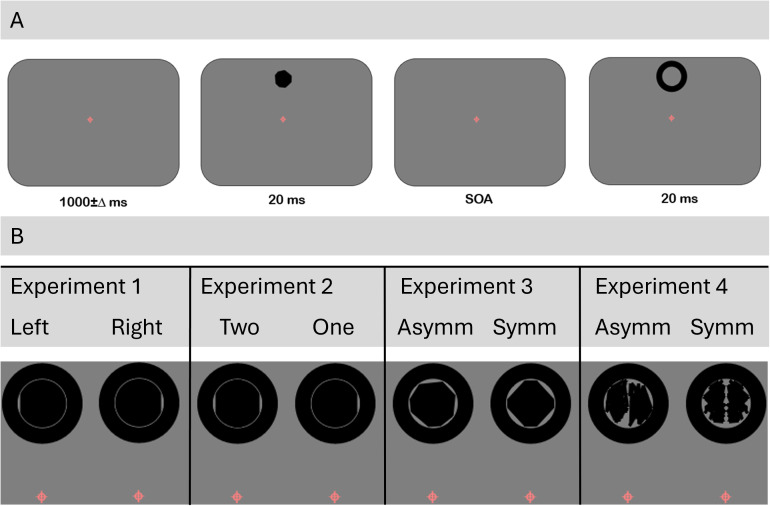
A: Schematic of the trial sequence. The target (e.g., disk or shape) was shown for 20 ms, followed by the mask with stimulus onset asynchronies (SOAs) ranging from 0 to 200 ms. The mask, an annular ring, did not overlap with the target and was displayed for 20 ms. Participants responded after the mask disappeared. Eye-tracking ensured that fixation remained within a ± 1° boundary around a red fixation cross, located 3° below the target. B: Schematic of stimuli across the four experiments. Stimuli were positioned 3° above fixation in all experiments.

Experiment 4: Abstract Irregular Shapes (Symmetry Discrimination). Abstract, irregular shapes were generated by connecting 10 randomly placed vertices within a 1.4° circular boundary. In the symmetric condition, one half of the shape was mirrored along a vertical axis, while in the asymmetric condition, the vertices were placed independently on both sides. All final stimuli possessed at most one axis of vertical symmetry. As in Experiment 3, the mask surrounded the shape with a separation of ~0.5° from its outermost points. Participants judged whether the shape was symmetrical or asymmetrical. Stimuli for each experiment are shown in Fig 1B.

### Participants

We estimated the statistical power to detect a significant quadratic trend—indicative of Type B masking—using 1,000 simulations implemented in the simr package [59] in R. The simulations were based on pilot data from six participants, each completing 400 trials with the same stimuli and timing parameters used in Experiment 1. Effect size and variability were derived from these pilot datasets, and orthogonal contrasts were specified using the emmeans package [60]. The simulation results indicated that achieving 90% power to detect a quadratic SOA effect would require approximately 1,440 mask trials. Given that each participant in the main experiment completed 180 mask trials and 200 total trials (including 20 no-mask trials), this corresponded to a minimum sample size of eight participants per experiment.

To accommodate potential exclusions due to poor task engagement or insufficient perceptual sensitivity, we increased the initial sample size to 18 participants per experiment. Participants whose accuracy in the no-mask baseline condition fell below 65% were excluded from analysis. This cutoff was chosen because, under a binomial distribution, 65% represents the minimum accuracy required to be significantly above chance (p < .05) given the 20 no-mask trials. This criterion ensured that participants contributed interpretable data and demonstrated adequate engagement with the task, thus providing a valid baseline for assessing the impact of masking.

The same participant group (14 females, 4 males; mean age = 21 years, SD = 0.97) completed Experiments 1, 2, and 4, while a separate group (10 females, 8 males; mean age = 22 years, SD = 1.45) was recruited for Experiment 3. After applying the exclusion criterion, the final sample sizes were: Experiment 1: 12 participants; Experiment 2: 9 participants; Experiment 3: 15 participants; Experiment 4: 14 participants.

Data collection began on April 24, 2024, and concluded on June 28, 2024. All participants provided written informed consent prior to participation. The study was conducted in accordance with the Declaration of Helsinki and approved by the Ethics Committee of the University of Padova (Protocol 413-b, Area 17).

### Data analysis

All experiments employed a binary forced-choice paradigm, but the nature of the decision varied by task. In Experiment 1, participants indicated whether the missing segment was on the left or right side of a disk-shaped target. In Experiment 2, they judged whether the target was missing one or two segments. In Experiments 3 and 4, participants classified shapes as either symmetric or non-symmetric. On each trial, accuracy was coded as a binary variable: 1 for correct and 0 for incorrect, based on the match between the participant’s response and the trial’s correct classification.

We analysed binary accuracy data (correct/incorrect) using generalized linear mixed-effects models (GLMMs), which are well-suited for modelling repeated categorical outcomes in hierarchical data structures. GLMMs were selected for three main reasons: (1) the dependent variable was binary (correct/incorrect), (2) the data featured repeated measures nested within participants, and (3) individual variability in baseline performance needed to be modelled via random effects. Each model was estimated using the lme4 package in R [61] with a binomial distribution and logit link function. The model formula was specified as: Accuracy ~ SOA + (1 | Participant) where Accuracy was a trial-level binary variable (1 = correct, 0 = incorrect), SOA was treated as an ordered factor, and Participant was entered as a random intercept to account for within-subject correlations.

Importantly, we did not collapse or average data across trials or participants prior to model fitting. Each trial contributed to the estimation of model parameters, allowing us to capture both fixed effects of SOA and random inter-individual variability.

To evaluate the overall influence of SOA on accuracy, we conducted Type III Wald chi-square tests using the Anova() function from the car package [62]. This omnibus test assessed whether accuracy differed across SOA levels, analogous to a repeated-measures ANOVA but within the GLMM framework.

When the omnibus test indicated a significant SOA effect, we conducted planned post hoc comparisons between each SOA level and the no-mask baseline. These comparisons were based on estimated marginal means (EMMs) computed via the emmeans package [60], EMMs represent model-based predictions of accuracy for each SOA, adjusted for all other model parameters and averaged over random effects. These were back-transformed from the logit scale to provide interpretable estimates of probability of correct response (i.e., percentage accuracy). Multiple comparisons were corrected using the False Discovery Rate (FDR) procedure.

In addition to pairwise contrasts, we examined the functional form of the masking function by applying orthogonal polynomial contrasts to the SOA factor. These included:

- Linear trends, indicating a monotonic change in accuracy across SOAs.
- Quadratic trends, which test for the canonical inverted U-shape (Type B masking).
- Cubic and quartic trends, capturing more complex or asymmetric masking profiles.

Each contrast isolates variance uniquely attributable to that polynomial component, allowing us to determine whether the classic quadratic Type B shape sufficed or whether additional curvature was required to model the data adequately.

For data visualization, we plotted observed mean accuracy at each SOA by averaging binary accuracy scores across all valid trials per SOA, along with standard errors. These visualizations served as intuitive summaries of group-level performance but were distinct from the inferential statistics derived from the GLMM.

To assess differences in masking depth across tasks, we compared the difference between the no-mask baseline and the minimum accuracy SOA in each experiment. These differences were tested across experiments using two-sample Z-tests, incorporating standard errors derived from the observed means and bootstrapped SEs. This enabled us to quantify whether tasks requiring explicit symmetry processing (Experiments 3 and 4) showed stronger or earlier suppression than those involving simpler geometric shapes (Experiments 1 and 2), thus probing whether feedback-dependent integration modulates masking profiles.

## Results

### Experiment 1: Shape discrimination task (left or right missing segment)

**Fig 2:**
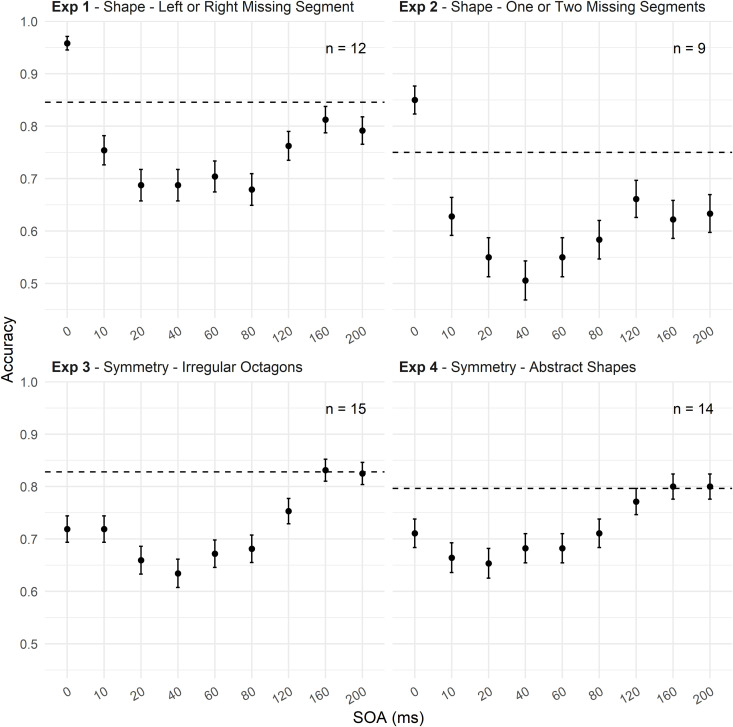
Accuracy across SOA conditions in Experiments 1 to 4. Experiment 1 (Shape Discrimination Task) shows an inverted U-shaped masking curve, with facilitation at 0 ms SOA and suppression at intermediate SOAs (10–80 ms). Accuracy recovers at longer SOAs (160–200 ms). The dashed line represents the no-mask performance baseline. Experiment 2 (Shape Discrimination Task: One vs. Two Missing Segments) replicates a similar inverted U-shaped function. Facilitation is observed at 0 ms SOA, with significant suppression at intermediate SOAs and recovery at longer SOAs. Experiment 3 (Symmetry Task with Octagonal Shapes) reveals a reduction in accuracy, particularly at 0 ms SOA, with an inverted U-shaped curve at intermediate SOAs. Experiment 4 (Symmetry Task with Abstract Irregular Shapes) shows no facilitation at 0 ms SOA. A sharp drop in accuracy occurs at short SOAs, with a gradual recovery at longer SOAs. Error bars represent standard error (SE).

Six participants were excluded for failing to meet the criteria (data visualized in Fig 2). The omnibus test revealed a significant main effect of SOA, χ²(8) = 101.87, p < 0.001. A significant linear trend, z = −3.298, p = 0.001, indicated decreased accuracy with shorter SOAs. The quadratic trend, z = 7.500, p < 0.001, suggested an inverted U-shaped relationship characteristic of metacontrast masking. Additionally, a significant cubic trend, z = −5.214, p < 0.001, and further higher-order trends (fourth-order: z = 2.056, p = 0.040; fifth-order: z = −2.851, p = 0.004) indicated nonlinear variations in accuracy across SOAs.

Post-hoc comparisons revealed significant facilitation at 0 ms SOA, where accuracy increased by 11.2% compared to the no-mask baseline (84.6%), z = 4.438, p < 0.001. As expected, accuracy decreased at intermediate SOAs (10 ms to 80 ms, all p < 0.001), and recovered at longer SOA (160 ms), where no significant difference from baseline was observed. This pattern reflects classic metacontrast masking (Table 1).

**Table 1:** Post-hoc comparisons of SOA conditions to the no-mask baseline (Experiment 1).

SOA	Probability	SE	df	z-ratio	p-value
0	0.958	0.0129	Inf	4.438	<.0001
10	0.754	0.0278	Inf	−3.878	0.0002
20	0.688	0.0299	Inf	−6.562	<.0001
40	0.688	0.0299	Inf	−6.562	<.0001
60	0.704	0.0295	Inf	−5.905	<.0001
80	0.679	0.0301	Inf	−6.887	<.0001
120	0.762	0.0275	Inf	−3.533	0.0005
160	0.812	0.0252	Inf	−1.427	0.1537
200	0.792	0.0262	Inf	−2.311	0.0234

### Experiment 2: Implicit symmetry discrimination task (one or two missing segments)

Nine participants were excluded (data visualized in Fig 2). The omnibus test revealed a significant main effect of SOA, χ²(8) = 65.927, p < 0.001. A significant linear trend, z = −2.709, p = 0.007, indicated decreased accuracy with shorter SOAs. The quadratic trend, z = 6.023, p < 0.001, suggested an inverted U-shaped relationship between SOA and accuracy. A significant cubic trend, z = −4.866, p < 0.001, also indicated nonlinear accuracy patterns across SOAs.

The no-mask baseline accuracy was 75.0%. At a SOA of 0 ms, a facilitation effect of 10% was observed (z = 3.047, p = 0.0026), indicating improved performance compared to the no-mask baseline. Significant accuracy suppression occurred at all SOAs (all p < 0.01), with the minimum accuracy recorded at a SOA of 40 ms, which was 50.6% (see Table 2).

**Table 2:** Post-hoc comparisons of SOA conditions to the no-mask baseline (Experiment 2).

SOA	Probability	SE	df	z-ratio	p-value
0	0.850	0.0266	Inf	3.047	0.0026
10	0.628	0.0360	Inf	−3.735	0.0003
20	0.550	0.0371	Inf	−5.993	<.0001
40	0.506	0.0373	Inf	−7.220	<.0001
60	0.550	0.0371	Inf	−5.993	<.0001
80	0.583	0.0367	Inf	−5.041	<.0001
120	0.661	0.0353	Inf	−2.733	0.0063
160	0.622	0.0361	Inf	−3.900	0.0002
200	0.633	0.0359	Inf	−3.569	0.0005

### Experiment 3: Symmetry discrimination task (octagonal shapes)

Three participants were excluded (data visualized in Fig 2). The omnibus test revealed a significant main effect of SOA, χ²(8) = 65.266, p < 0.001. A significant linear trend, z = 5.317, p < 0.001, and quadratic trend, z = 5.360, p < 0.001, indicated an inverted U-shaped pattern typical of metacontrast masking. The quartic trend was also significant, z = −2.245, p = 0.024. Higher-order trends were not significant.

The no-mask baseline accuracy was 82.8%. At 0 ms SOA, a significant 10.9% drop in accuracy (z = −5.100, p < 0.001) was observed, with continued suppression at SOAs between 20 ms and 80 ms (all p < 0.001). Accuracy recovered at 160 ms and 200 ms, with no significant difference from baseline (Table 3).

**Table 3:** Post-hoc comparisons of SOA conditions to the no-mask baseline (Experiment 3).

SOA	Probability	SE	df	z-ratio	p-value
0	0.719	0.0251	Inf	−5.100	<.0001
10	0.719	0.0251	Inf	−5.100	<.0001
20	0.659	0.0265	Inf	−7.731	<.0001
40	0.634	0.0269	Inf	−8.799	<.0001
60	0.672	0.0262	Inf	−7.187	<.0001
80	0.681	0.0260	Inf	−6.776	<.0001
120	0.753	0.0241	Inf	−3.525	0.0005
160	0.831	0.0209	Inf	0.148	0.8822
200	0.825	0.0212	Inf	−0.148	0.8822

### Experiment 4: Symmetry discrimination task (abstract irregular shapes)

Four participants were excluded (data visualized in Fig 2). The omnibus test revealed a significant main effect of SOA, χ²(8) = 37.003, p < 0.001. Linear (z = 4.981, p < 0.001) and quadratic trends (z = 2.799, p = 0.005) were significant, indicating increased accuracy with longer SOAs. Higher-order trends were not significant.

The no-mask baseline accuracy was 79.6%. Significant suppression occurred at SOAs from 0 ms to 80 ms (all p < 0.001). Performance recovered at longer SOAs (160 ms and 200 ms), with no significant differences from baseline (Table 4).

**Table 4:** Post-hoc comparisons of SOA conditions to the no-mask baseline (Experiment 4).

SOA	Probability	SE	df	z-ratio	p-value
0	0.711	0.0271	Inf	−3.530	0.0006
10	0.664	0.0282	Inf	−5.387	<.0001
20	0.654	0.0284	Inf	−5.807	<.0001
40	0.682	0.0278	Inf	−4.679	<.0001
60	0.682	0.0278	Inf	−4.679	<.0001
80	0.711	0.0271	Inf	−3.530	0.0006
120	0.771	0.0251	Inf	−1.038	0.3848
160	0.800	0.0239	Inf	0.148	0.8820
200	0.800	0.0239	Inf	0.148	0.8820

### Comparison across experiments

To assess how task demands, and stimulus structure modulate masking dynamics, we extracted and compared three key behavioural indices across the four experiments:

(1) Facilitation at 0 ms SOA, defined as the difference in accuracy between the 0 ms SOA condition and the no-mask baseline, indicating potential perceptual integration or grouping.
(2) Maximum inhibition, calculated as the largest drop in accuracy relative to the baseline, corresponding to the SOA at which masking was most effective.
(3) Optimum SOA, defined as the temporal point at which performance reached its minimum (i.e., the “dip” of the U-shaped masking function), indexing the latency of peak suppression.

Pairwise Z-tests on the facilitation measure revealed no significant difference between Experiment 1 and Experiment 2 (Z = –0.251, p = 0.802), suggesting similar facilitation magnitudes when the stimuli were simple and spatially complementary. However, robust differences emerged when comparing Experiments 1 and 2 to the symmetry-based tasks in Experiments 3 and 4. Specifically, significant reductions in facilitation were observed between Experiment 1 and Experiment 3 (Z = –5.24, p < 0.001), Experiment 1 and Experiment 4 (Z = –4.40, p < 0.001), Experiment 2 and Experiment 3 (Z = –3.93, p < 0.001), and Experiment 2 and Experiment 4 (Z = –3.35, p < 0.001).

In contrast, the maximum inhibition effect—the depth of the performance dip—was comparable across all experiments, despite differences in the temporal dynamics as reported in Table 5.

**Table 5:** Masking Metrics Across Experimental Conditions.

Experiment	Facilitation 0 ms SOA	Maximum Inhibition	Optimum SOA (Dip)
Exp. 1: Shape (Segment)	+11.2%	–16.7%	80 ms
Exp. 2: Symmetry (Segments)	+10.0%	–24.4%	40 ms
Exp. 3: Symmetry (Octagons)	–10.9%	–19.4%	40 ms
Exp. 4: Symmetry (Abstract)	–8.5%	–14.2%	20 ms

## Discussion

The present study investigated whether symmetry perception is susceptible to metacontrast masking and how task demands shape the temporal profile of suppression. Across all four experiments, we observed a robust quadratic trend in the accuracy-by-SOA function, consistent with the classical inverted U-shaped profile of Type B masking. Suppression peaked at intermediate SOAs and diminished at both earlier and later intervals, supporting interference with feedback-dependent processing. Significant higher-order polynomial trends were also observed, suggesting subtle deviations from a strictly parabolic masking profile. These inflections may reflect differences in task complexity, perceptual load, or individual variability. Although our study was not powered to model such fine-grained features, future curve-fitting approaches (e.g., Gaussian or Weibull modelling) could better characterize latency shifts and asymmetries in masking functions.

Our findings reveal that facilitation at 0 ms SOA is not uniformly present but varies systematically with the perceptual relationship between target and mask and the demands of the task. In Experiment 1, which directly replicated the stimulus configuration employed by Aydin et al. (2021) [3], the combination of a central disk target and an annular mask elicited robust facilitation at 0 ms SOA. Experiment 2 involved subtle symmetry variations using one- or two-segment disks and retained some facilitation. However, in Experiments 3 and 4—where target and mask were more complex, non-complementary, and required explicit symmetry judgments—facilitation disappeared, and suppression emerged at the earliest SOA.

Facilitation at 0 ms SOA in Experiments 1 and 2 likely reflects successful perceptual grouping, driven by structural congruency between target and mask. Target and mask were spatially separated, but when they formed a complementary configuration—such as a disk with a missing segment enclosed by an annular mask—they enabled grouping and enhanced visibility. This resonates with Gestalt principles of good continuation and closure, where the visual system organizes elements into coherent wholes based on alignment and completeness. Crucially, such facilitation is not merely the absence of masking, but a consequence of object formation processes, as shown by experiments where the mask shape and not the target shape was manipulated. Sayim, Westheimer, and Herzog (2010) [31], demonstrated that performance in vernier-offset discrimination improved when flanking elements were arranged into perceptually coherent shapes. Their study manipulated only the configuration of mask-like elements, keeping the target constant—essentially the inverse of our approach—but arrived at a similar conclusion: that perceptual grouping can mitigate interference. This cross-paradigm convergence suggests that whether facilitation is achieved depends less on task format and more on the presence of spatial coherence sufficient to trigger object-level representations. Thus, both our findings and those of Sayim et al. support the notion that coherent grouping can protect against perceptual degradation, whether through masking or contextual crowding.

When the target’s missing segment aligns with the mask’s inner contour, the resulting “gap” can act as a perceptual figure in its own right—highlighted not by luminance contrast, but by its topological status as an enclosed absence. In this configuration, the visual system treats the hole as a salient internal structure, a pop-out feature that facilitates rapid grouping and object completion [63,64]. This interpretation suggests that facilitation at 0 ms SOA may be driven not only by contour continuity but also by the salient “hole-ness” of the completed figure—a higher-order property that guides early organization and resists suppression.

This interpretation aligns with Bachmann and Allik’s (1976) [65] and Francis and Cho’s (2008) [4] proposals that temporal integration contributes to early facilitation. When target and mask are presented simultaneously, the visual system may fuse them into a single percept. If spatially coherent, this fusion supports target identification; if not, it impairs it. Thus, the masking function reflects not just timing but also how spatial and temporal properties jointly influence integration. Herzog and Koch (2001) [66] also demonstrated that perception at very short SOAs reflects emergent properties of target–mask configurations, which may yield integration-based facilitation rather than suppression under favourable spatial arrangements.

Facilitation in our study was also likely enhanced by the topological salience of the stimuli. According to the “global-first” hypothesis [63,67], the visual system prioritizes topological invariants—such as holes—over local features. Stimuli with coherent, hole-like structures benefit from early grouping. Supporting this, Meng et al., (2012) [68] found that such configurations dominate perception in continuous flash suppression, and Bertamini & Lawson (2006) [64] demonstrated that holes enhance figure–ground segmentation. Similarly, Duangudom et al. (2007) [69] emphasized that spatial layout, rather than low-level stimulus energy, determines the type of masking observed. In our study, such layout-based coherence appears to have supported early integration and facilitated performance in Experiments 1 and 2, whereas its absence in Experiments 3 and 4 led to early suppression.

By contrast, when the target was irregular or structurally incompatible with the mask (Experiments 3 and 4), facilitation was absent and masking emerged from the earliest SOA. These results closely parallel the transition described by Maksimov et al. (2011) [70], who found that congruent target–mask pairs produce Type B masking (non-monotonic, with facilitation at short SOAs), whereas incongruent pairs yield Type A functions, marked by monotonic suppression. Our own data replicate this transition: perceptual coherence fosters facilitation; its absence elicits immediate interference.

Computational modelling results further bolster this interpretation. Rüter et al. (2011) [71] demonstrated that dynamic models of masking fail to account for empirical data unless they incorporate mechanisms for perceptual grouping. In simulations of the shine-through paradigm—where a vernier target is followed by a temporally separated grating mask—they showed that model accuracy depends not only on SOA but on the spatial structure of the stimuli. Only when the model included grouping principles could it replicate human performance, especially the facilitation observed under conditions of spatial coherence. Perceptual outcomes in masking are determined not merely by temporal parameters or by low-level features like luminance, but also by figure–ground relationships and the global configuration.

This conclusion is corroborated by behavioural findings from Sayim et al. (2014) [72], who investigated the role of Gestalt grouping in backward masking using a vernier discrimination task. They varied the spatial arrangement, colour homogeneity, and regularity of mask elements while keeping basic physical parameters constant. Masks that formed coherent Gestalts (e.g., uniform colour and regular spacing) produced significantly weaker masking than irregular or heterogeneous ones. The results could not be explained by simple feature-based interactions and instead pointed to higher-level perceptual segmentation processes that mitigate interference when the visual system succeeds in grouping target and mask. In this framework, grouping serves as a protective mechanism: when target and mask are perceived as part of a single object, suppression is minimized; when they compete for segmentation, suppression increases.

Our own findings extend this framework by showing that the structure of target–mask configurations modulate masking even when low-level stimulus properties are held constant. In Experiments 1 and 2, structural alignment and perceptual coherence facilitated integration, likely supported by feedback mechanisms. In Experiments 3 and 4, grouping was disrupted, and masking emerged at the earliest SOAs. This pattern mirrors findings in crowding, where the effectiveness of flankers in interfering with target perception depends on whether they are grouped into a coherent structure. Recent theoretical frameworks suggest that such perceptual organization is not immediate or automatic, but depends on temporally extended processing mediated by recurrent feedback. Doerig et al. (2020) [12] provided compelling evidence for this claim by comparing human performance to that of standard deep convolutional neural networks (DCNNs) in crowding tasks. While humans showed improved performance when global configurations enabled grouping, DCNNs—which rely exclusively on feedforward processing—failed to exploit global structure. This dissociation highlights the essential role of recurrent, feedback-supported integration in perceptual organization. Grouping, in this view, is not a passive byproduct of visual encoding but an active process unfolding over time through bidirectional communication between higher-order and lower-order visual areas.

Our results reveal a systematic progression in suppression dynamics across experimental conditions. Specifically, the stimulus onset asynchrony (SOA) at which masking was most effective—that is, where accuracy reached its minimum—shifted progressively earlier from Experiment 1 through Experiment 4: suppression peaked at 80 ms in Experiment 1, at 40 ms in Experiments 2 and 3, and at 20 ms in Experiment 4. This leftward shift in the suppression dip presents an apparent contradiction with predictions derived from both the dual-channel model [73] and recurrent processing theory [16].

According to the dual-channel account, visual masking arises from temporal interference between two parallel feedforward processing streams: a fast, transient magnocellular channel and a slower, sustained parvocellular channel [18,26]. This model predicts that visual features processed primarily via the parvocellular system—such as contour and brightness—exhibit distinct temporal masking profiles due to their differential processing speeds. Specifically, contour discrimination tasks should be most strongly disrupted at shorter metacontrast stimulus onset asynchronies (SOAs), typically around 30–50 ms, whereas brightness judgments should show peak masking at somewhat later SOAs, between 50 and 80 ms, reflecting the slower development of surface information in the sustained channel [13,28,73]. In contrast, reentrant models emphasize the temporally extended, recursive nature of visual processing, positing that the conscious perception of complex visual properties—such as global shape, figure–ground segmentation, or symmetry—depends on feedback loops from higher-order visual areas to early sensory cortices [16,32]. From this perspective, tasks requiring feedback-supported integration should be particularly vulnerable to backward masking at later SOAs, typically beyond 80 ms, when reentrant activity is still unfolding and susceptible to disruption by subsequent visual input [24,74].

In our findings the tasks presumed to rely most heavily on reentrant processing—Experiments 3 and 4, which involved explicit judgments of bilateral symmetry—exhibited suppression peaks at the earliest SOAs tested. This was unexpected; however, one possibility is that, in the absence of compatibility between target and mask, the visual system does not integrate the two. Without successful integration, the masking function becomes more symmetrical, and the suppression dip occurs earlier. This interpretation is consistent with the absence of facilitation at SOA = 0 ms in these experiments, and with the immediate onset of suppression at minimal temporal offsets.

Importantly, even in Experiments 3 and 4, the characteristic U-shaped profile of Type B masking remains preserved, with suppression that could extend up to 120 ms (as seen in Experiment 3). This observation suggests that early suppression does not truncate the full temporal dynamics of masking, but instead reflects a shift in the timing of peak interference. Rather than an absence of reentrant processing, the shift may imply that feedback mechanisms are engaged earlier or that early feedforward interactions become more vulnerable to interference. In either case, the persistence of a Type B pattern indicates that crosstalk between channels and feedback processes are still operating, but that the balance between early feedforward and later reentrant processes is altered.

It is also plausible that the observed leftward shift in suppression peaks is at least partly attributable to sampling variability or limitations in SOA resolution. Given the smooth nature of the masking curves and the 20 ms sampling intervals, finer-grained SOA increments may be necessary to more precisely determine the exact location of the suppression dip. For this reason, we refrain from drawing definitive conclusions regarding changes in the temporal architecture of visual processing.

Nevertheless, the trend remains theoretically meaningful. It suggests that the temporal trajectory of masking is shaped not only by SOA or task difficulty, but by the structural coherence of the stimuli and their capacity to support perceptual grouping. When the visual system succeeds in forming a unified object representation—via spatial alignment or topological cues—feedback processes may help sustain perceptual stability, delaying the onset of suppression. When coherence is lacking, early feedforward interactions may dominate, and masking arises sooner. In this view, perceptual organization shapes not just whether masking occurs, but when and how strongly it unfolds across time.

Neurophysiological data reinforce this temporal and hierarchical account of visual masking. Kraut & Albrecht (2022) [11] identified ERP markers of perceptual segmentation in masking—such as contour integration negativity (CIN)—and showed that suppression is modulated by the phase of ongoing cortical oscillations, linking masking strength to both pre-stimulus neural readiness and post-stimulus feedback. Likewise, Catak et al. (2024) [75] demonstrated that attentional load modulates ERP components associated with visual awareness, thereby influencing masking strength. Together, these findings suggest that masking is not a purely stimulus-driven phenomenon but one shaped by cognitive resource allocation and the temporal window of perceptual evidence accumulation.

Tasks involving the detection of global visual structures—such as symmetry—are likely initiated by early, rapid processing stages but require prolonged temporal integration windows, typically spanning 300–500 milliseconds, for perceptual evidence to accumulate and stabilize [16]. This temporally extended processing reflects the necessity of recurrent feedback loops operating across the ventral visual stream to bind spatially distributed features into unified, coherent representations [76]. In particular, the perception of symmetry under non-trivial or noisy conditions relies heavily on these recurrent dynamics, as symmetry-related integration necessitates iterative binding mechanisms that exceed the computational scope of immediate feedforward extraction [37,46,48]. These findings resonate with broader theoretical frameworks of visual consciousness, which posit that phenomenal awareness emerges from recurrent, large-scale cortical interactions rather than from isolated feedforward sweeps [77].

Disruption within this temporal window—particularly by metacontrast masking—interferes with the reentrant loops required for perceptual stabilization [20]. Boehler et al. (2008) [52] and Carlson et al. (2007) [51] found that masking reduces activation in LOC and V4, areas critical for symmetry perception. Lamme et al. (2002) [53] demonstrated that masking abolishes feedback from higher-tier ventral areas to V1, halting global perceptual consolidation. Importantly, this disruption is not independent of feedforward processing: recurrent processing is dynamically intertwined with the initial feedforward sweep [16,76]. Any interruption to early input can undermine the feedback cycles that follow, disrupting the transition from preconscious processing to awareness. Nakashima et al. (2024) [78] extend this framework developmentally, showing that conscious access in children likewise depends on recurrent loops building upon intact early visual input. Thus, the neural architecture responsible for symmetry detection overlaps with regions most susceptible to feedback disruption, and the effects of masking reflect a breakdown of this interaction, providing a mechanistic explanation for the pronounced Type B masking profiles observed in Experiments 3 and 4.

Our findings refine the dual-route model of masking [18,79–81], which posits that fast feedforward processing via the magnocellular (transient/dorsal) pathway precedes slower feedback in the parvocellular (sustained/ventral) stream. In our paradigm, the abrupt-onset mask likely engaged the dorsal stream, while the target—especially under symmetry conditions—was primarily processed in the ventral stream. Feedforward crosstalk from the M-pathway disrupts slower P-pathway processing, particularly at intermediate SOAs (50–80 ms), consistent with our suppression peaks.

These results challenge earlier claims that symmetry perception is immune to masking [55]. While symmetry can be extracted rapidly under optimal conditions, our findings show it remains vulnerable to temporal interference—especially when tasks rely on feedback-supported integration. The consistent Type B masking profiles across all tasks suggest that suppression can result from intrachannel disruption within the ventral stream, even in the absence of dorsal–ventral competition.

Finally, these findings carry both methodological and theoretical implications. Experimental designs that promote perceptual grouping—such as the classic disk-and-annulus configuration—can complicate the interpretation of neural facilitation effects at early SOAs (e.g., 0 ms), particularly when contrasted with suppression at later delays (e.g., 50 ms). This concern was already acknowledged by Aydin et al. (2021) [3], who observed performance enhancement at both 0 and 10 ms SOAs in their behavioural pre-study. To reduce the likelihood of target–mask integration in their EEG recordings, they deliberately excluded the 0 ms condition and instead compared 10 and 50 ms SOAs. As noted in their Methods section, the 10 ms SOA was selected as the shortest interval that could still limit perceptual overlap. However, performance at 10 ms remained approximately 7% above the no-mask baseline, suggesting that some residual integration may have persisted despite this precaution.

Future neuroimaging studies should carefully disentangle perceptual grouping from masking effects when analysing early neural signatures of awareness. This is particularly important in light of recent ERP findings by Sztuka & Kühn (2024) [82], who reported that late-stage suppression in symmetry tasks under perceptual load is accompanied by fronto-parietal disengagement and a reduction in P3 amplitude. These results suggest that higher-order cortical contributions to perceptual organization and awareness may be disrupted when early visual processing is compromised—such as in masking paradigms—highlighting the importance of late integrative stages for conscious access.

## Conclusion

Our study demonstrates that metacontrast masking is dynamically modulated by stimulus structure, task demands, and the availability of feedback. Facilitation at 0 ms SOA does not merely signal an absence of suppression but reflects successful perceptual grouping and structural integration between target and mask. Symmetry detection, which relies on spatially distributed integration supported by temporally extended reentrant processing, is particularly vulnerable to early interruption. These feedback mechanisms, essential for constructing coherent global representations, may initiate earlier and persist longer when external grouping cues are absent, thus expanding the window of masking susceptibility.

In line with Lamme’s recurrent processing framework [16] and the global neuronal workspace theory Dehaene & Changeux (2011) [76], we propose that conscious access to symmetrical structure emerges only when feedback cycles are allowed to complete. Premature disruption of these cycles—whether through masking or other temporal interference—impairs the transition from sensory encoding to perceptual awareness. Our findings reinforce the interdependence of feedforward and feedback dynamics, showing that early disruptions cascade through recurrent loops, particularly when global structure must be integrated.

Together, these results support a dynamic, interactionist model of masking, in which suppression arises from both intra- and inter-channel interference, modulated by the perceptual requirements of the task. This interpretation aligns with contemporary models of recurrent visual computation and points to promising directions for future work—especially studies using EEG, MEG, and fMRI to dissect the neural timing and topography of global integration under masking. Understanding the full interplay between stimulus structure, task demands, and the temporal architecture of perceptual systems remains central to advancing theories of conscious vision.

### Declaration of generative AI and AI-assisted technologies in the writing process

During the preparation of this work, the authors used ChatGPT 4.0 (OpenAI) to improve the readability and language of the manuscript. After using this tool, the authors reviewed and edited the content as needed. The authors take full responsibility for the content of the published article.
